# Randomized single-blind comparative study of the midazolam/pethidine combination and midazolam alone during bronchoscopy

**DOI:** 10.1186/s12885-022-09640-y

**Published:** 2022-05-12

**Authors:** Masahiro Katsurada, Motoko Tachihara, Naoko Katsurada, Naoya Takata, Hiroki Sato, Chihiro Mimura, Junya Yoshioka, Koichi Furukawa, Masako Yumura, Takehiro Otoshi, Yuichiro Yasuda, Tatsunori Kiriu, Daisuke Hazama, Tatsuya Nagano, Masatsugu Yamamoto, Yoshihiro Nishimura, Kazuyuki Kobayashi

**Affiliations:** Division of Respiratory Medicine, Department of Internal Medicine, Kobe University Graduate School of Medicine, 7-5-1 Kusunoki-cho, Chuo-ku, Kobe, 650-0017 Japan

**Keywords:** Bronchoscopy, Lung cancer, Midazolam, Pethidine, Sedation

## Abstract

**Background:**

Bronchoscopy can be a distress for the patient. There have been few studies on the combination of sedatives and opioids. The aim of this study was to demonstrate the usefulness and safety of administration of the combination of midazolam and pethidine during bronchoscopy.

**Methods:**

In this prospective randomized single (patient)-blind study, we randomly assigned 100 patients who were scheduled to undergo bronchoscopy biopsy to receive treatment with either the midazolam/pethidine combination (combination group) or midazolam alone (midazolam group) during examinations. After the end of bronchoscopy, patients completed a questionnaire and the visual analogue scale was measured. The primary outcome was the patients’ acceptance of re-examination assessed by visual analogue scale. We also assessed pain levels, vital signs, midazolam use, xylocaine use, and adverse events. Univariate analyses were performed using Fisher’s exact test for categorical data, and the t-test or Mann-Whitney test was carried out for analysis of numeric data. All *P*-values were two-sided, and values < 0.05 were considered statistically significant.

**Results:**

We analyzed 47 patients in the combination group and 49 patients in the midazolam group. The primary outcome was a good trend in the combination group, but not significantly different (3.82 ± 2.3 in combination group versus 4.17 ± 2.75 in midazolam alone, *P* = 0.400). In the combination group, the visual analog scale score for pain during bronchoscopy was significantly lower (1.10 ± 1.88 versus 2.13 ± 2.42, *P* = 0.022), and the sedation level score per the modified observer’s assessment of alertness/sedation scale was significantly deeper (3.49 ± 0.98 versus 3.94 ± 1.03, *P* = 0.031). Maximal systolic blood pressure during testing was significantly lower (162.39 ± 23.45 mmHg versus 178.24 ± 30.24 mmHg, *P* = 0.005), and the number of additional administrations of midazolam was significantly lower (2.06 ± 1.45 versus 2.63 ± 1.35, *P* = 0.049). There were also significantly fewer adverse events (30 versus 41, *P* = 0.036).

**Conclusions:**

The combination uses of midazolam and pethidine for sedation resulted in significant improvements in the pain, blood pressure, additional use of midazolam, and safety during bronchoscopy among patients.

**Trial registration:**

This study was registered in the University Medical Hospital Information Network in Japan (UMINCTR Registration number: UMIN000032230, Registered: 13/April/2018).

**Supplementary Information:**

The online version contains supplementary material available at 10.1186/s12885-022-09640-y.

## Background

Lung cancer has a high prevalence and is one of the leading causes of cancer death worldwide [1]. In the 2020 United States national epidemiology study, lung cancer was the highest proportion of deaths among different cancer types [2]. Advances in anti-lung cancer therapies have resulted in prolonged 5-year survival rates for lung cancer patients [3]. In cases of positivity for driver oncogene mutations, the use of selective tyrosine kinase inhibitor (TKI) can lead to a dramatic increase in survival rates [4]. When lung cancer becomes resistant to the first TKI, other TKIs can be effective for specific resistance genes. Therefore, the re-examination of driver oncogenes is very important.

Flexible bronchoscopy is one of the most common lung cancer tissue sampling methods. To examine acquired resistance genes, repeated bronchoscopy is also necessary. However, the bronchoscopy itself is very distressful for the patient. So, the reduction of distress is very important. The British Thoracic Society guideline recommend the use of a combination of opioids and midazolam [5]. According to a Japanese nationwide survey of bronchoscopy, medications were selected in 76.9% of cases with midazolam, 10.9% with pethidine, 4.4% with fentanyl, 4.2% with propofol, and 3.6% with others [6]. Thus, opioids are used by only 15% of the population, and pethidine is more often used than fentanyl. Pethidine an opioid receptor agonist with central analgesic effects. Its analgesic effects is one-fifth to one-tenth of morphine. Compared with morphine, pethidine causes less urinary retention and constipation and less respiratory depression. The plasma half-life is 3 to 4 hours. If injected rapidly, respiratory suppression, hypotension, circulatory disturbance may occur [7]. Fentanyl is a high potency (50 to 100 fold higher than morphine) synthetic opioid with potent analgesic properties. Immediately after administration, it produces profound analgesia to external stimuli, as well as respiratory depression, bradycardia, and other morphine-like effects. The onset of action is rapid and the duration of action is short, ranging from 30 minutes to 1 hour, but it accumulates progressively with repeated administration [8]. Midazolam exerts hypnotic, sedative, anxiolytic, amnesic, anticonvulsant, and muscle relaxant effects by activating gamma amino butyric acid receptors, which are inhibitory neurotransmitters in the central nervous system. When midazolam is used, respiratory depression, tongue depressions, and hypotension should be noted. If oversedation occurs, flumazenil, an antagonist, should be administered. A retrospective study looking at the effect of adding pethidine or fentanyl to midazolam in intranasal ultrathin bronchoscopy showed no difference in safety between the two groups [9].

There have been few studies on the combination of sedatives and opioids. The best sedative and opioid combinations are unknown and there have been no randomized reports on the usefulness of midazolam plus pethidine in bronchoscopy. The aim of this study was to demonstrate the usefulness and safety of administration of the combination of midazolam and pethidine during bronchoscopy.

## Methods

In this prospective single-center, randomized, single (patient)-blind study, we compared midazolam alone and the midazolam/pethidine combination during bronchoscopy. The aim of this study was to demonstrate the usefulness and safety of administration of the combination of midazolam and pethidine during bronchoscopy.

### Patients

Patients between 20 and 79 years of age were eligible for enrollment if they were scheduled to undergo bronchoscopy for biopsy of lung tumors or mediastinal tumors at Kobe University Hospital. Patients with any one of the following criteria were excluded: a) allergy to the drugs used in this study and drug hypersensitivity, b) acute narrow angle glaucoma, c) myasthenia gravis, d) uncontrollable bronchial asthma, e) severe respiratory depression confirmed before the examination, f) heart failure secondary to chronic lung disease, h) convulsive state, i) severe liver functional failure, j) pregnancy, potential pregnancy or lactation, k) taking HIV protease inhibitors or monoamine inhibitors, l) use of orally administered opioids when performing bronchoscopy, and m) judgment of being inappropriate for inclusion by the staff in charge of this examination.

### Trial design and treatment

This study used block randomization to evenly allocate the midazolam/pethidine combination (combination group) or midazolam alone (midazolam group). Randomization was not stratified. Patients undergoing bronchoscopy first took 5 mL of 2% xylocaine syrup by mouth and lay in a supine position for 5 minutes. After the patient spat out the syrup, 5 mL of 2% xylocaine liquid was sprayed into the patient’s throat. The patient lay on the examination table and was attached to a vital sign mechanical monitor, including an electrocardiogram waveform monitor, a pulse oximeter and a blood pressure monitor. The combination group received 2 mg/2 mL midazolam (Astellas, Tokyo in Japan) and 17.5 mg/5 mL pethidine (Takeda, Tokyo in Japan) intravenously. The midazolam group received 2 mg/2 mL midazolam and 5 mL saline as a placebo intravenously. Patients aged 75 to 79 years or weighing less than 45 kg were given half the dose of midazolam and pethidine. Oral bronchoscopy intubation was started 3 minutes after the intravenous injection. When the sedation level was considered shallow, an additional 1 mg/1 mL of midazolam could be administered intravenously at an interval of more than 4 minutes per the preference of the operator. When the patient’s cough was severe, 1 mL of 2% xylocaine was administered into the trachea via a bronchoscopic approach. Vital sign monitoring and depth of sedation were checked by the assisting doctor every 2.5 minutes. The types of bronchoscopes were 1TQ290, 1 T260, F260, P290, P260, and MP290 (Olympus, Tokyo in Japan) for peripheral and central lesions, and were UC290F and UC260FW (Olympus, Tokyo in Japan) for endobronchial ultrasound-guided transbronchial needle aspiration (EBUS-TBNA). If the patient was over sedated at the end of bronchoscopy, 0.25 mg/2.5 mL flumazenil was administered intravenously, and 0.25 mg was mixed with saline infusion and administered in drops. When patients were aware and conscious 1 hour after the end of the bronchoscopy, they filled out a questionnaire about their level of pain. We defined adverse events related to bronchoscopy as any adverse event that occurred up to 1 week after bronchoscopy. This study was approved by the ethics committee of Kobe University (300023) and was conducted in accordance with the Helsinki declaration. Written informed consent was provided voluntarily by the patient before enrollment. This study was registered in the University Medical Hospital Information Network in Japan (UMINCTR Registration number: UMIN000032230, Registered: 13/April/2018).

https://upload.umin.ac.jp/cgi-bin/icdr/ctr_view_reg.cgi?recptno=R000036694

### Trial assessments

The questionnaire on patient distress during bronchoscopy consisted of ten questions: i) Did you have any concerns before the test? ii) Was the throat anesthesia you had before the bronchoscopy painful? iii) Do you remember what happened during the bronchoscopy? iv) Did you feel distressed during the bronchoscopy? v) Did you experience any pain during bronchoscopy? vi) Did you have difficulty breathing during the bronchoscopy? vii) Did you have a cough during the bronchoscopy? viii) Did you feel like the examination took a long time? ix) How are you feeling after the test? and x) Do you think you could have another bronchoscopy if necessary? Questions 1 to 10 were rated on a continuous scale from 0 (good) to 10 (bad) by means of the visual analog scale (VAS). We also included a yes or no answer to question 10 (Additional file 1). Sixty minutes after the end of bronchoscopy, patients completed a questionnaire.

The depth of sedation was assessed by means of the modified observer’s assessment of alertness/sedation scale (MOAA/S scale) [10] (Additional file 2). The target depth was scores 3 or 4.

In addition to the above items, we assessed the following: sex, age, body weight, height, the amount of 2% xylocaine administered into the trachea through a bronchoscope, total midazolam dosage, vital signs (heart rate (HR), blood pressure (BP), oxygen saturation (SpO_2_)), targeted tumor size (short diameter of lymph nodes, long diameter of other targeted lesion), examination time, the types of bronchoscopes, the type of technique, diagnosis, and adverse events. Systolic hypertension was defined as over 180 mmHg, and hypoxemia was defined as SpO_2_ under 90%. Tachycardia was defined as over 130 beats per minute.

### Outcomes

Given the possibility that the patient may have another bronchoscopy in the future, the primary outcome was the patients’ acceptance of re-examination assessed by VAS of the questionnaire, “Question 10″. The secondary outcomes included the percentage of patients who responded that they would undergo reexamination by bronchoscopy, the VAS for each question, the dosages of xylocaine and midazolam and safety.

### Randomization

This study is a prospective single-center, randomized, single (patient)-blind study. The study used block randomization to evenly allocate the intervention and control groups. Block sizes of 2 or 4 were randomly created. An allocation physician who was not involved in the bronchoscopy created a random function in Microsoft Excel software, assigned a block size of 2 or 4 to each random function, and created an allocation form. The allocation sheets were maintained in a password-protected file that could not be viewed by any other researcher. The researcher sent the ID of the patient undergoing bronchoscopy to the allocation manager. The allocation manager typed the patient IDs into a randomized allocation form in the order in which they were sent and communicated to the bronchoscopist whether the patient was in the intervention or control group. During the examination, pethidine was administered as CodeA and placebo (saline) as CodeB, so that the patients did not know which pethidine or placebo they were receiving.

### Statistical analysis

Since the effect of the midazolam plus pethidine combination therapy is unknown, the estimated number of patients was calculated based on previous single group comparative studies of sedative drugs and single group comparative studies of opioids [11, 12] and the number of accumulative cases over 3 years at Kobe University. We required 45 cases in each group when calculating the mean difference in the VAS score between the two groups, the midazolam alone and combination groups, assuming a mean difference of 1.94 and a standard deviation of 3.24 and assuming a two-sided significance level of 5% and a t-test at 80% power. Assuming 10% dropout, a total of 50 cases in each group were estimated to be the required number of cases.

Categorical data are reported as numbers (percentages), and numeric data are reported as means ± standard deviations. Univariate analyses were performed using Fisher’s exact test for categorical data, and the t-test or Mann-Whitney test was carried out for analysis of numeric data. All *P*-values were two-sided, and values < 0.05 were considered statistically significant. All statistical analyses were performed with EZR version 1.51 (http://www.jichi.ac.jp/saitama-sct/SaitamaHP.files/statmed.html; Kanda, 2020), a graphical user interface for R (The R Foundation for Statistical Computing, Vienna, Austria, version 3.6.3 ) [13].

## Results

### Patient characteristics and preoperative vital signs

**Fig. 1:**
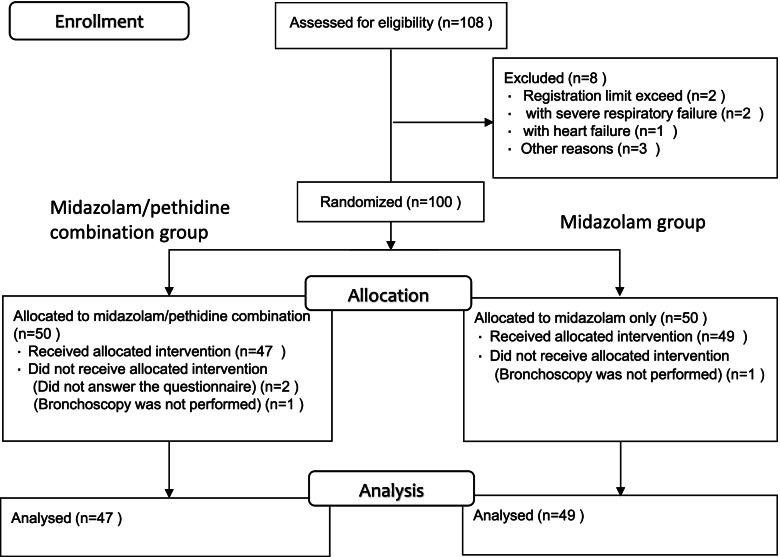
CONSORT Flow Diagram

A total of 100 patients were enrolled between June 4, 2019, and July 31, 2020. In the combination group, 2 patients did not answer the question about the primary outcome, and 1 patient did not undergo bronchoscopy. In the midazolam group, 1 patient did not undergo bronchoscopy. We analyzed 47 patients in the combination group and 49 patients in the midazolam group (Fig. 1).

There were no significant differences in patient characteristics, except for height. There were no significant differences in target lesions. There was no significant difference in the preoperative systolic blood pressure or SpO_2_ (Table 1).

**Table 1:** Patient characteristics and preoperative vital signs

	Combination group	Midazolam group	
	*N =* 47	*N =* 49	*p*.value
Sex, No. (%)			
Male	33 (70.2)	34 (69.4)	1.000
Female	14 (29.8)	15 (30.6)	
Age	66.28 ± 9.57	68.14 ± 9.12	0.330
Height (cm)	164.46 ± 8.33	160.96 ± 8.38	†0.043
Weight (kg)	58.58 ± 11.67	59.63 ± 11.95	0.666
BMI (kg/m^2^)	21.52 ± 3.15	22.98 ± 4.18	0.057
Long diameter of target lesion (mm)	33.23 ± 15.47	36.00 ± 18.38	0.542
Short diameter of lymph node (mm)	21.00 ± 6.97	17.25 ± 6.23	0.083
Location of a lesion (%)			
bronchus	1 (2.1)	1 (2.0)	0.411
right upper lobe	4 (8.5)	5 (10.2)	
right middle lobe	2 (4.3)	2 (4.1)	
right lower lobe	9 (19.1)	4 (8.2)	
left upper lobe	7 (14.9)	7 (14.3)	
left lower lobe	9 (19.1)	6 (12.2)	
hilar lymph node	5 (10.6)	4 (8.2)	
mediastinal lymph node	10 (21.3)	20 (40.8)	
Preoperative oxygen saturation	98.26 ± 1.70%	98.27 ± 1.71%	0.977
Preoperative systolic blood pressure	139.35 ± 19.64 mmHg	141.00 ± 19.89 mmHg	0.686

### The types of bronchoscopic procedures

We performed bronchial wash, endobronchial biopsy, transbronchial biopsy (TBB), EBUS-GS-TBB, conventional transbronchial needle biopsy (TBNA), and EBUS-TBNA. Between the two groups, there were no significant differences in the types of bronchoscopic procedures (Table 2).

**Table 2:** The types of bronchoscopic procedures

	Combination group	Midazolam group	*p*.value
	*N =* 47	*N =* 49	
Primary procedure (%)			
bronchial wash	1 (2.1)	0 (0.0)	0.210
endobronchial biopsy	12 (25.5)	5 (10.2)	
transbronchial biopsy	2 (4.3)	3 (6.1)	
EBUS-GS-TBB	16 (34.0)	17 (34.7)	
conventional TBNA	2 (4.3)	1 (2.0)	
EBUS-TBNA	14 (29.8)	23 (46.9)	
Additional procedure (%)			
bronchial wash	0 (0.0)	1 (2.0)	1.000
brush	0 (0.0)	1 (2.0)	
curretage	0 (0.0)	1 (2.0)	
transbronchial biopsy	1 (2.1)	1 (2.0)	
EBUS-GS-TBB	1 (2.1)	0 (0.0)	

*EBUS-GS-TBB* Endobronchial ultrasonography with a guide sheath transbronchial biopsy

*TBNA* Trans-bronchial needle aspiration

*EBUS-TBNA* Endobronchial ultrasound-guided trans-bronchial needle aspiration

### VAS of patients’ experiences of pain before and during bronchoscopy

There was no significant difference regarding the primary outcome (VAS for question 10: patients’ acceptance of re-examination) (3.82 ± 2.93 in the combination group vs. 4.17 ± 2.75 in the midazolam group, *P* = 0.400).

The VAS was highest for question 1 (patients’ concerns before the test) in both groups (5.62 ± 3.28 vs. 5.40 ± 2.94, *P* = 0.721). The VAS for question 4 (patients’ distress during the bronchoscopy) tended to be better in the combination group (2.48 ± 2.80 vs. 3.46 ± 3.00, *P* = 0.103). The VAS was significantly better (1.10 ± 1.88 vs. 2.13 ± 2.42, *P* = 0.022) in the combination group for question 5 (patients’ pain during bronchoscopy) (Table 3).

**Table 3:** Visual Analog Scale of patients’ subjective pain before and during bronchoscopy

	Combination group	Midazolam group	*p.value*
	*N* = 47	*N* = 49	
Q1. Did you have any concerns before the test?	5.62 ± 3.28	5.40 ± 2.94	0.721
Q2. Was the throat anesthesia you had before the test painful?	3.92 ± 2.94	3.86 ± 2.74	0.907
Q3. Do you remember what happened during the inspection?	4.03 ± 3.96	4.77 ± 3.65	0.340
Q4. Did you feel distressed during the examination?	2.48 ± 2.80	3.46 ± 3.00	0.103
Q5. Did you experience any pain during the bronchoscopy?	1.10 ± 1.88	2.13 ± 2.42	†0.022
Q6. Did you have difficulty of breathing during the bronchoscopy?	2.25 ± 2.85	2.84 ± 2.82	0.319
Q7. Did you have a cough during the bronchoscopy?	3.01 ± 2.92	3.43 ± 3.12	0.494
Q8. Did you feel like the examination took a long time?	3.69 ± 2.39	3.61 ± 2.32	0.869
Q9. How are you feeling after the test?	3.40 ± 2.19	3.70 ± 2.05	0.487
Q10. Do you think you could have another bronchoscopy if necessary?	3.82 ± 2.93	4.17 ± 2.75	0.547
Q11. Do you think you could have another bronchoscopy if necessary? (yes or no)			0.400
yes	38 (80.9)	35 (71.4)	
no	9 (19.1)	14 (28.6)	

### Objective indicators and vital signs

In the combination group, the examination time was significantly shorter (30.55 ± 8.08 minutes vs. 34.73 ± 7.71 minutes, *P* = 0.011), and the additional number of midazolam administrations was significantly lower (2.06 ± 1.45 vs. 2.63 ± 1.35, *P* = 0.049). Total amount of midazolam (3.83 ± 1.56 mg vs. 4.27 ± 1.34 mg, *P* = 0.145) and the amount of 2% xylocaine treatments also tended to be lower in the combination group (18.26 ± 3.97 mL vs. 19.70 ± 3.67 mL, *P* = 0.076). Maximal systolic blood pressure during testing was significantly lower (162.39 ± 23.45 mmHg vs. 178.24 ± 30.24 mmHg, *P* = 0.005), and hypoxemia tended to be better in the combination group (92.13 ± 3.25% vs. 90.69 ± 5.31%, *P* = 0.116). The lowest sedation score, MOAA/S, was significantly deeper (3.49 ± 0.98 vs. 3.94 ± 1.03, *P* = 0.031) in the combination group (Table 4).

**Table 4:** Objective indicators and vital signs

	Combination group	Midazolam group	*p.value*
	*N* = 47	*N* = 49	
Examination time (minute)	30.55 ± 8.08	34.73 ± 7.71	†0.011
Initial dosage of midazolam (mg)	1.77 ± 0.43	1.69 ± 0.47	0.432
Number of additional administration of midazolam	2.06 ± 1.45	2.63 ± 1.35	†0.049
Total dosage of midazolam (mg)	3.83 ± 1.56	4.27 ± 1.34	0.145
Total dosage of 2% xylocaine (mL)	18.26 ± 3.97	19.70 ± 3.67	0.076
Maximal systolic blood pressure (mmHg)	162.39 ± 23.45	178.24 ± 30.24	†0.005
Highest heart rate (bpm)	104.38 ± 16.86	105.59 ± 17.81	0.734
Lowest MOAA/S scale	3.49 ± 0.98	3.94 ± 1.03	†0.031
Lowest oxygen saturation (%)	92.13 ± 3.25	90.69 ± 5.31	0.116
Usage of flumazenil (%)			0.983
Yes	1 ( 2.1)	0 ( 0.0)	
No	46 (97.9)	49 (100.0)	

*MOAA/S scale* Modified Observer’s Assessment of Alertness/Sedation Scale

### Adverse events and final diagnosis

**Fig. 2:**
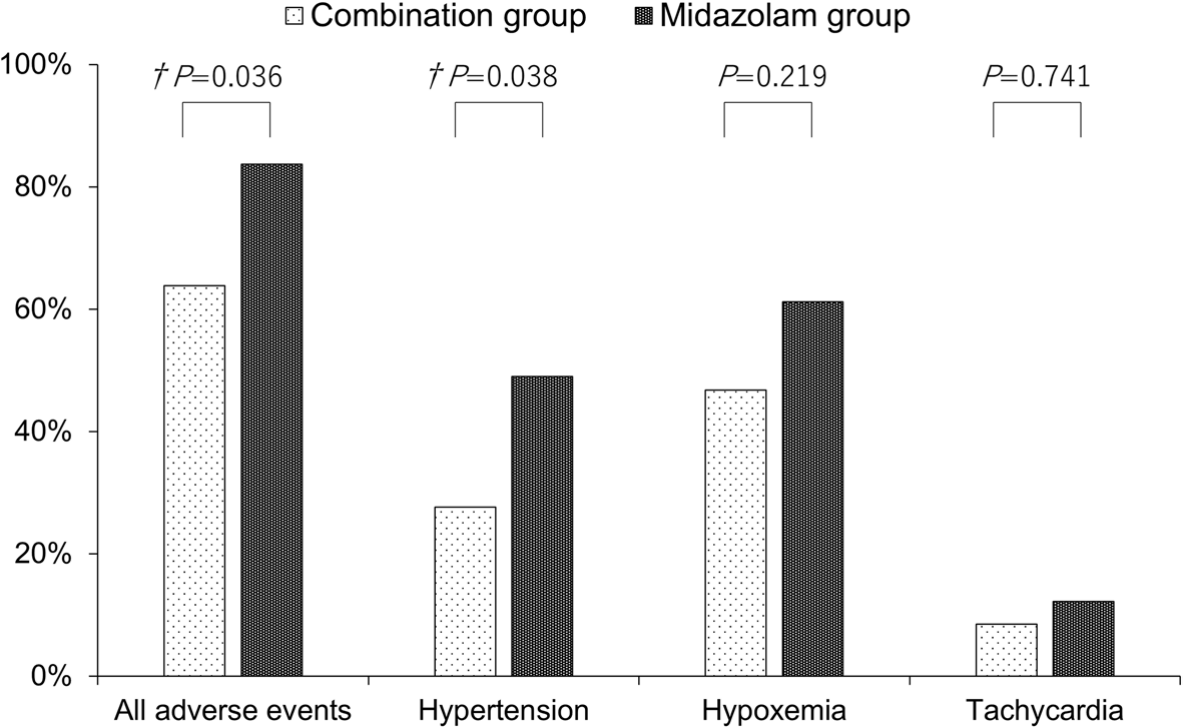
Adverse events

All adverse events were significantly more frequent in the midazolam group (30 in the combination group vs. 41 in the midazolam group, *P* = 0.036). Hypertension was significantly more frequent in the midazolam group (13 in the combination group vs. 24 in the midazolam group, *P* = 0.038). Hypoxemia was not different in both group (22 in the combination group vs. 30 in the midazolam group, *P* = 0.219) (Fig. 2). In particular, severe complications such as pneumothorax and stroke were observed in the midazolam group. The most frequent adverse event was hypoxemia, followed by hypertension (Additional file 3). The final diagnoses were not significantly different between the two groups (Additional file 4).

## Discussion

This study is the first randomized comparative study on the benefit of sedation during bronchoscopy in combination with pethidine and midazolam.

In previous trials that compared placebo versus sedatives, the benefit was greater in the group that received diazepam [14, 15]. In a comparative study of propofol and midazolam, propofol reached the desired degrees of sedation and awakening more quickly than midazolam [16, 17], had no significant difference in the risk of hypoxemia [18]. Propofol may be useful, but unlike midazolam, there is no antagonist medication against oversedation.

In a comparative study of sedatives versus opioids during bronchoscopy, alfentanil and midazolam were compared. Alfentanil reduced cough, but patients’ distress was lower with midazolam [19]. This study showed that opioids reduced coughing, and midazolam decreased patient’s consciousness.

About the study comparing sedation and sedative/opioid combinations during bronchoscopy, there was one randomized, double blind, placebo-controlled study of midazolam only and midazolam/hydrocodone combination. This study showed that patients’ cough and discomfort scores were better in the combination group and that the mean lowest SpO_2_ under supplemental oxygen was similar in both groups [20]. Another small cohort study comparing midazolam alone with the midazolam plus alfentanil combination reported improvements in patient distress VAS and a significant reduction in the midazolam dose in the combination group. There was no group difference in minimum SaO_2_ and heart rate [12]. They are important papers on the combined use of opioids and sedatives. However, the analysis was performed for bronchoalveolar lavage or transbronchial lung biopsy. It is necessary to evaluate the combination of opioids and sedatives in bronchoscopic procedures now that EBUS-GS and EBUS-TBNA have become mainstream.

In a single-arm study of bronchoscopy with midazolam and fentanyl, patients’ VAS scores for consent to retest were reported to be significantly improved. The procedures in this study were TBB, bronchial brushing, bronchial washing, and EBUS-TBNA [21]. This study showed the usefulness of the combination of midazolam and fentanyl in EBUS-TBNA and TBB. However, because it was a single-arm study, it was difficult to compare safety. There is few evidence on which sedatives and opioid are best to use.

There are several randomized studies reporting on the use of pethidine or fentanyl in combination with sedatives in gastrointestinal endoscopy. In endoscopic retrograde cholangiopancreatography, a randomized comparative trial of propofol plus pethidine versus propofol plus fentanyl was conducted. In this trial, completion rates, time to procedure completion, length of stay in the recovery room, respiratory and cardiovascular complications, and patient satisfaction and operator satisfaction were similar [22]. In upper gastrointestinal endoscopy, there are two randomized studies and one prospective observational study comparing midazolam with pethidine or fentanyl. In these two randomized controlled trials, examination time, patient satisfaction, and incident cases were similar in both groups [23, 24]. In a prospective observational study, the duration of stay in the recovery room was shorter in the fentanyl group [25]. In colonoscopy, there is a showed no significant differences in safety and operator satisfaction between the fentanyl and pethidine groups [24, 26]. Patient satisfaction was significantly higher in the pethidine group [24]. These studies show no clear safety difference in endoscopy between the pethidine plus sedation group and the fentanyl plus sedation group. Since pethidine is used more frequently than fentanyl in Japan, our study was conducted using pethidine [6].

In our study, there was a significant improvement in subjective symptoms of pain during testing in the midazolam-pethidine combination group. There was a tendency for the midazolam pethidine combination group to be better in VAS for patient distress and coughing, but there was no significant difference. There was no significant difference in the percentage of patients willing to be retested. The reason for the lack of difference in the patient’s subjective symptoms is the possibility that the patient’s memory during the examination might have been blurred due to midazolam and that the patient may have been prepared to undergo the examination with some patience if necessary. Therefore, considering the retrograde amnesia associated with midazolam, it is possible that the endpoint of “Do you think you could have another bronchoscopy if necessary?” was inappropriately set.

We found that the combination with pethidine resulted in a deeper sedation, a significant reduction in examination time, and a significant reduction in the number of midazolam additions. Given that a MOAA/S score of 3 to 4 has been studied as an appropriate sedative score for the MOAA/S score [27], it is likely that the combination group was more appropriately sedated. Due to adequate sedation, the increase in systolic blood pressure during the test could be significantly suppressed. There were significantly fewer intra- and post-test adverse events in the midazolam pethidine group. There was also less hypoxemia during testing in the pethidine combination group. Given that there was only one case of oversedation, it is likely that the cause of the hypoxemia was cough, and the concomitant use of pethidine reduced the cough and lowered the risk of hypoxemia. Particularly important, pneumothorax and cerebral infarction were observed in the midazolam alone group. These results indicate that the combination of pethidine and midazolam produces good results on objective indicators such as depth of sedation, vital signs during the examination and adverse events.

There are several limitations in this study. The first is that it was a single-blind study. To assess patients’ subjective symptoms, patients were not told whether they were in the combination or midazolam group. Thus, the primary endpoint was assessed by patient questionnaire responses, so bias mediation is unlikely, but it is possible that midazolam-induced retrograde biogenic amnesia could have influenced the study results. We believe that the universality of this study is assured, as objective indicators such as vital signs, test time and adverse events have improved. The second point is that the MOAA/S score was used in the assessment of depth of sedation. Although the MOAA/S score is neatly scored, the MOAA/S score is assessed by the assistant doctor and may be subjectively biased by the doctor. It would have been better if we could have conducted a double-blinded study to evaluate the ease of examination for the operator, assistants, and nurses. The use of bispectral index monitoring may also be useful in future studies, as it is a more objective assessment of sedation. The third point is that this study was a single-center study, and the number of patients was relatively small. The reason for choosing a single-center study was to prevent variability in assessment by limiting the number of evaluators and to allow for rapid response to emergencies by experiencing a large number of cases. We believe it is necessary to conduct this multicenter study because this single-center study has shown the benefit of the combination of midazolam and pethidine. The fourth point is that we do not know if midazolam and pethidine are the best combination. The combination of pethidine and midazolam has been shown to be useful in endoscopic pharyngeal observation [28]. However, there is also a report showing the benefit of remimazolam in bronchoscopic sedation [27] and a report showing the benefit of the combination of dextometetodine [29]. In the field of anesthesia other than endoscopy, the usefulness of sufentanil and clonidine combinations has also been noted [30, 31]. Fentanyl is also frequently used in bronchoscopy. So, the best combination remains to be determined. To resolve these problems, we are currently conducting a randomized, double-blind, comparative study of midazolam alone versus midazolam plus fentanyl in EBUS-TBNA.

## Conclusions

In bronchoscopic sedation, the combination of midazolam and pethidine attenuated pain and showed significant improvements in objective indicators such as vital signs and adverse events during the examination.

## Additional files

**Additional file 1.** Questionnaire.

**Additional file 2.** Modified Observer’s Assessment of Alertness/Sedation Scale.

**Additional file 3.** Adverse events during and after bronchoscopy.

**Additional file 4.** Diagnosis of biopsy.

## Data Availability

The datasets used and/or analyzed during the current study are available from the corresponding author on reasonable request.

## Abbreviation

BP: Blood pressure
EBUS-GS-TBB: Endobronchial ultrasonography with a guide sheath transbronchial biopsy
EBUS-TBNA: Endobronchial ultrasound-guided transbronchial needle aspiration
HR: Heart rate
MOAA/S scale: Modified observer’s assessment of alertness/sedation scale
VAS: Visual analog scale
TBB: Transbronchial biopsy
TBNA: Transbronchial needle biopsy
SpO_2_: Oxygen saturation
TKI: Tyrosine kinase inhibitor
