# Supplementary material for: Randomized single-blind comparative study of the midazolam/pethidine combination and midazolam alone during bronchoscopy
Source: BMC Cancer. 2022 May 12;22:539. doi: 10.1186/s12885-022-09640-y (PMC9102220; doi:10.1186/s12885-022-09640-y)
Supplement: Supplementary file 1 — Additional file 1. Questionnaire. [file 12885_2022_9640_MOESM1_ESM.docx]

Additional file 1. Questionnaire

**①Did you have any concerns before the test?**

**😊No　　　　　　　　　　　　　　　　 　　　　 Yes😢**

**②** **Was the throat anesthesia you had before the bronchoscopy painful?**

**😊No 　　　　　　　　　　　　　　　　　 Yes😢**

**③** **Do you remember what happened during the bronchoscopy?**

**😊No　　　　　　　　　　　　　　　　　　　　 Yes😢**

**④** **Did you feel distressed during the bronchoscopy?**

**😊No　　　　　　　　　　　　　　　　　　　　 Yes😢**

**⑤ Did you experience any pain during the bronchoscopy?**

**😊No　　　　　　　　　　　　　　　　　　　　 Yes😢**

**⑥** **Did you have difficulty of breathing during the bronchoscopy?**

**😊No　　　　　　　　　　　　　　　　　　　　 Yes😢**

**⑦** **Did you have a cough during the bronchoscopy?**

**😊No　　　　　　　　　　　　　　　　　　　　 Yes😢**

**⑧ Did you feel like the bronchoscopy took a long time?**

**😊Feel short　　　　　　　　　　　 　　　　　　　　　　　Feel long😢**

**⑨** **How are you feeling after the bronchoscopy?**

**😊Good　　　　　　　　　　　　　　　 　　　　　　Bad😢**

**⑩** **Do you think you could have another bronchoscopy if necessary?**

**😊Yes, I accept 　　　　　　　　　　　　　　　　　　 NO, I reject.😢**

**Yes　　　　No**
